# Clinicopathological Spectrum of Diabetic and Non-diabetic Renal Lesions in Patients with Diabetes Mellitus: An Experience from A Tertiary Care Center

**DOI:** 10.30699/ijp.2024.2024462.3270

**Published:** 2024-07-24

**Authors:** Netra Prakash Kori, Manjunath Revanasiddappa, Nagraj D Naik, Atul Desai, Ranjana Shashidhar Ranade

**Affiliations:** 1 Department of Pathology, SDM College of Medical Sciences and Hospital, Shri Dharmasthala Manjunatheshwara University, Dharwad, Karnataka, India; 2 Department of Nephrology, SDM College of Medical Sciences and Hospital, Shri Dharmasthala Manjunatheshwara University, Dharwad, Karnataka, India; 3 Department of Pathology, KLE JGMMMC, Hubballi, a unit of KLE Academy of Higher Education and Research, Belagavi, India

**Keywords:** Diabetes Mellitus, DN, NDRD, Renal biopsy, CKD

## Abstract

**Background & Objective:**

Diabetic patients often develop lesions called non-diabetic renal diseases (NDRD), whose prognostic and therapeutic implications vary from diabetic nephropathy (DN). Since early identification of NDRD is associated with a better prognosis, we aimed to understand its spectrum.

**Methods:**

One hundred and thirty-four patients were included in a cross-sectional study. Their clinical, and laboratory data and indications for biopsy were recorded. Two cores of renal tissue were studied under light microscopy and immunofluorescence; patients were classified into NDRD, NDRD+DN, and DN groups.

**Results:**

Of all the patients studied, five were diagnosed with type 1 diabetes mellitus (DM1), and the rest were type 2 diabetes mellitus (DM2). Overall, the male-to-female ratio was 3:1. The Mean age of NDRD patients was the greatest, and males were predominant in all three groups. CKD was the most common presentation. Except for significantly greater proteinuria and hematuria in the DN and NDRD groups respectively, others were not different. Infection-related glomerulonephritis was the most common lesion among the NDRD+DN group, whereas IgA nephropathy and acute tubulointerstitial nephritis were frequent in the NDRD group.

**Conclusion:**

Based on our findings, renal biopsy should be considered in all those diabetic patients with lesser onset duration presenting with hematuria, no/minimal retinopathy, and minimal proteinuria. A precise diagnosis helps in providing timely therapy for NDRD and prolonging renal and patient survival.

## Introduction

The prevalence of diabetes mellitus (DM), a global epidemic (1), is increasing. Around 20-40% of these patients develop diabetic kidney disease (DKD)- diabetic nephropathy (DN). Other forms of nephropathy unrelated to DM, called non-diabetic renal disease (NDRD), are either isolated or superimposed on DN (2). They are more common in DM2 [12%–80% prevalence as compared to 2%–3% in type 1 diabetes mellitus (DM1)] and affect 26.7% of the Asian population (3).

Clinically, NDRD is characterized by abrupt proteinuria, absence of neuropathy and retinopathy, hematuria, and a rapid decline in renal function (4). Some of these in addition to the age of onset and duration of DM predicted its development in DM2 (5-8). Precise diagnosis of the glomerular and tubulo-interstitial lesions requires a renal biopsy, however, patients and clinicians might be averse to do biopsy, given its associated risks (9). Moreover, as it is relatively contraindicated in a single kidney and small kidneys (10) diagnoses will have to be based solely on the clinical and laboratory parameters, prompting a good understanding of the spectrum of NDRD.

Unlike DN, NDRD is reversible and often has a good prognosis when identified early (3). Given the different prognostic and therapeutic implications, we aimed to evaluate the clinical, laboratory, and renal biopsy features of diabetic and non-diabetic renal lesions in patients with DM.

## Material and Methods

The study was cross-sectional and descriptive. We included all the renal biopsy cases of diabetic patients presenting with renal involvement to the Department of Pathology, SDM College of Medical Sciences and Hospital, from Jan 2018 to December 2021. Inadequate biopsy specimens (<8 glomeruli), transplant kidney biopsies, and tissues not available for immunofluorescence were excluded. Our objectives were i) To estimate clinical and laboratory characteristics of diabetic patients presenting with renal involvement and ii) To classify these patients as diabetic nephropathy (DN) and non-diabetic renal disease (NDRD) or an overlap (DN+NDRD) based on the renal biopsy findings. 

Diagnosis of diabetic nephropathy based on the microscopic examination is characterized by the presence of glomerular basement membrane thickening. Other features include mesangial expansion, Kimmelstiel-Wilson nodules, microaneurysms, exudative lesions in the form of fibrin cap and/or capsular drop, and hyalinosis of both afferent and efferent arterioles (11).

Native renal biopsies of all DM patients were analyzed. Patients’ data regarding age, sex, co-morbidities, clinical presentation (edema, repeated infections), and renal syndromes (nephrotic, nephritic, AKI, RPGN) were collected. Blood pressure at admission was recorded. Urine examination findings, urine culture findings, serum creatinine, serum C3, C4, ANCA, Anti GBM, and absolute eosinophil count were documented. 

Two separate cores of renal tissue were analyzed under light microscopy (LM) and direct immunofluorescence (DIF) studies. For LM, the biopsy specimens were fixed in 10% buffered formalin, embedded in paraffin, and 3-4-microns thick sections were examined. Sections were stained with hematoxylin and eosin, PAS, silver methenamine, and Masson trichrome stain. DIF study was performed on the frozen sections using polyclonal antisera (FITC-conjugated Rabbit Antihuman Antisera manufactured by DAKO from Denmark) against human IgG, IgA, IgM, C3, C1q, kappa, and lambda light chains.

Renal Pathology Society Classification (11) was applied to categorize the patients into group 1= NDRD; group 2= NDRD superimposed over DN; and group 3= DN. DN lesions were further classified according to Tervaert* et al.*’s criteria (11) The NDRD lesions were subcategorized into glomerular, tubulo-interstitial, and vascular diseases. The degree of interstitial fibrosis, tubular atrophy, and interstitial inflammation were recorded.

The three groups were compared for their clinical and laboratory data using Chi-square analysis and unpaired t-test for categorical and continuous variables respectively. IBM SPSS software version 25 (SPSS Inc., Chicago, Ill., USA) was used for statistical analysis. A P-value of <0.05 was considered significant.

## Results

**Fig. 1:**
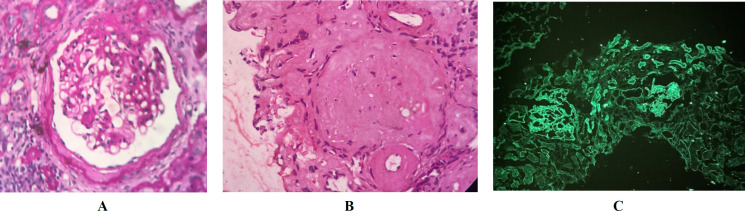
Characteristic light microscopic findings in diabetic nephropathy

Of the one hundred and thirty-four cases, the majority belonged to the NDRD+DN group, followed by isolated DN and isolated NDRD. All three groups had male predominance. Patients belonging to the isolated NDRD were older in comparison with other groups (Table 1). While almost half of them presented with CKD, AIN (Acute interstitial nephritis), AKI (acute kidney injury), and ATI/ATIN (Acute tubular injury/acute tubulointerstitial nephritis) were seen in 1% each (Figure 1).

A comparison of clinical and biochemical parameters is provided in Table 2. The number of patients with hematuria was the greatest in the NDRD group, while grades 3+ and 4+ proteinuria was found in a greater proportion of patients belonging to groups 2 and 3. The number of patients with retinopathy, low c3, and c4 and mean serum creatinine levels were not significantly different.

Histologic diagnosis for the patients in groups 1 and 2 is given in Table 3. Table 4 depicts the classification of DN patients. IgA Nephropathy and acute tubulointerstitial nephritis (3 cases each) were the most common renal biopsy findings in Group 1, whereas glomerulonephritis infection and acute tubulointerstitial nephritis were the most common renal lesions in Group 2.

**Table 1:** Descriptive statistics for the three groups

Group	No of cases n (%)	Mean age (years)
Group 1: Isolated NDRD	11 (8.2%); males-63.63%	56
Group 2: NDRD+ DN	62 (46.26%); males-85.48%	53
Group 3: Isolated DN	61 (45.52%); males- 67.21%	49

**Table 2:** Inter-group comparison of the clinical and biochemical parameters.

Clinical parameters	Group 1	Group 2	Group 3	P-value
Duration of DM	3 years ± 8 months (1mth-14yrs)	9yr ± 6 months (2-24yrs)	8 years (1mth-15y)	--
Retinopathy	3 cases (27.27%)	27 cases (43.54%)	27 cases (44.26%)	0.39
Proteinuria	2+ (70%) 3 cases 3+ to 4+ (27%)	49cases (79%) 3+ to 4+ (42%)	47 cases (77%) 3+ to 4+ (43%)	0.003
Hematuria	4 (36.33%)	7 (11%)	5 (8%)	0.009
*S. creatinine*	5.2	5.93	4.48	0.34
Hypertension	10 cases (90.1%)	52 cases (83.87%)	49 cases (80.32%)	--
Low C3 & C4	1(9%)	7 (11.29%)	14 (22.95%)	0.297

**Table 3:** Histological diagnosis for patients in Groups 1 and 2.

Histology	Group 1	Group 2
IgA nephropathy	3	1
AIN/ ATIN	3	11
ATI/ATN	2	11
MPGN	1	1
MGN	1	1
ANTI GBM crescentic	1	0
IRGN	-	12
Hypertension	-	7
CTIN	-	6
Pyelonephritis	-	2
FSGS	-	2
MCD	-	1
Granulomatous TIN	-	1
Malakoplakia	-	1

AIN/ATIN: Acute interstitial nephritis/Acute tubulointerstitial nephritis; ATI/ATN: Acute tubular injury/ Acute tubular necrosis. MPGN: Membranoproliferative glomerulonephritis; MGN: Membranous Nephropathy; IRGN: Infection-related glomerulonephritis; CTIN: Chronic tubulointerstitial nephritis; FSGS: Focal segmental glomerular sclerosis; MCD: Minimal change disease; TIN: Tubulointerstitial nephritis

**Table 4:** Sub-classification of DN patients in Group 3

DN classification	No of patients
Class 4 DN	45
Class 3 DN	11
Class 2A DN	2
Class 2B DN	3

A- Glomerulus, displaying K-W mesangial nodule in light microscopy. (PAS, x400)

B- Hyalinosis of afferent and efferent arterioles with sclerosed glomerulus in diabetic nephropathy. H and E, x400

C- Pseudolinear IgG along glomerular and tubular basement membrane in diabetic nephropathy (IgG antibody, DIF, X200)

**Fig. 2:**
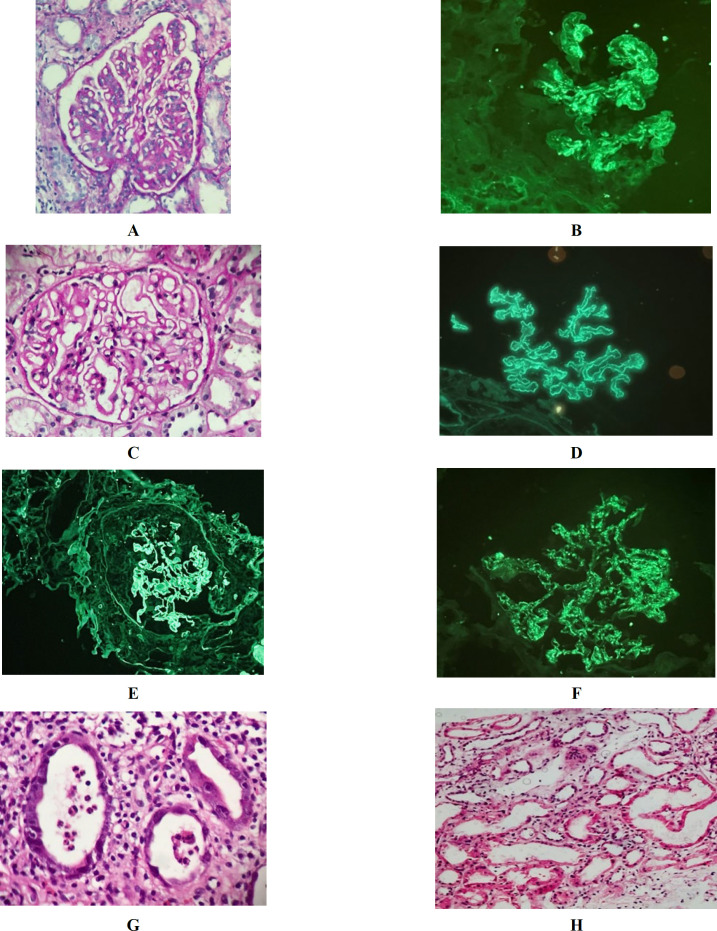
Characteristic renal biopsy findings in non-diabetic renal diseases; A- Diffuse mesangial proliferation in a case of IgA Nephropathy (PAS, X400)

B- Granular Mesangial IgA immune deposits in IgA Nephropathy (IgA antibody, DIF, X400)

C- Diffuse glomerular basement membrane thickening with perimembranous spikes in membranous nephropathy (PAS, X400)

D- Fine granular positivity with IgG antisera along the glomerular capillary wall in membranous nephropathy. (IgG antibody, DIF, X400)

E- Strong linear IgG deposits along the capillary wall in a case of anti-GBM Crescentic glomerulonephritis. Partial crescent is present in the glomerulus. (IgG antibody, DIF, X400)

F- Coarse granular positivity with C3 antisera in the mesangium and along the glomerular capillary wall in infection-related glomerulonephritis. (C3 antibody, DIF, X400)

G- Acute tubulointerstitial nephritis with occasional interstitial eosinophils. (H and E, X400)

H- Severe acute tubular injury in a case of diabetic nephropathy (H and E, X400)

## Discussion

Diabetic Nephropathy is present in approximately 40% of patients with diabetes for over 20 years and is a major cause of end-stage renal disease (ESRD) (10,12). Though it affects DM2 patients, there is often a spectrum of other renal diseases unrelated to diabetes called NDRD. The worldwide prevalence of NDRD varies from 12-80% (13). We detected a total prevalence of 54.4 % in our study- 8.2% for isolated NDRD and 46.2% for NDRD+DN. This was slightly lower than in previous Indian (1,7) and other studies (14-16), probably because we included both type 1 and 2 DM, whereas they recruited exclusively type 2 DM patients. Variation in incidence and prevalence rates from previous studies was attributed to factors like sample size, inclusion criteria, ethnicity, biopsy practices (4,17), and lack of availability of electron microscopy due to financial constraints and institutional limitations. Sirisha *et al.* (3) suggested that the actual prevalence of NDRD may sometimes be overestimated as renal biopsy is mostly performed for atypical clinical presentations.

Male predominance was noted in all three groups, in line with the findings of Kanodia* et al. *(1) and Yaqub* et al. *(18) which had a similar division of patients into NDRD, NDRD+DN, and DN. The mean age of NDRD patients was the highest (56 years), while DN patients were the youngest (mean age=49 years). This matches with several studies with younger DN patients (1,3,18). Mak* et al. *(5) reported an average age of 57 and 50 years for the DN and mixed lesions groups respectively. The above findings indicate that males in their fifth decade are more likely to be affected by NDRD.

Patients with exclusive DN lesions in our study had the longest duration of diabetes, although a significant difference was not detected. However, others (1,18) demonstrated a significantly greater duration in DN patients. Kritmetapak (19) concluded that a short duration of diabetes of <8 years is more likely to predict NDRD than DN. Results from studies by Kanodia* et al. *(1) and Kumar* et al. *(20) also denote that a shorter duration of diabetes is indicative of NDRD.

CKD was the most common clinical presentation, followed by sub-nephrotic proteinuria and AKI. Prakash* et al. *(6) reported CKD followed by acute nephritic syndrome as the most common presentation. Other studies by Sirisha* et al. *(3) identified RPRF and AKI; Das* et al. *(17) NS, RPRF, and AKI; Kanodia* et al. *(1) detected NS, AKI, and RPRF as being predominant. It is therefore clear that presenting symptoms can be quite different.

Histological diagnosis showed IgA nephropathy and AIN/ATIN in 3 each from group 1, while IRGN, ATI, AIN, and ATN were common in group 2. This is in line with Kanodia* et al. *(1) study of having detected ATIN (38.57%), benign nephrosclerosis (15.71%), and IgA nephropathy (7.14%) in most of their NDRD patients. Mahesh* et al. *(21) attributed increased AIN in NDRD to the significant use of antibiotics, proton pump inhibitors, and over-the-counter availability of analgesics. Sirisha* et al. *(3) observed that the IgA prevalence varied across the populations due to hereditary and racial predispositions for specific glomerulopathies. Glycation end products culminate into releasing proinflammatory cytokines and the oxidized low-density lipoproteins in diabetes cause immune-mediated inflammation, leading to the production of glomerular IgA deposits. IgA nephropathy superimposed on DN is commonly known and well-reported in the Indian, Chinese, and Asian populations. (3, 7, 22). However, rarely there might be a possibility of post-infectious glomerulonephritis superimposed on DN. In such instances, electron microscopy studies might demonstrate sub-epithelial humps and diagnosis may shift towards staphylococcal glomerulonephritis. However, none of these previous studies have included electron microscopy either. (1,2,3,4,6,7, 22). In our study, we encountered strong mesangial deposits of IgA along with lambda light chain predominance over kappa light chains (by 2+) in immunofluorescence for all cases of IgA nephropathy. In a study by Orfila C* et al. *(23), cases of IgA nephropathy with lambda light predominance were observed in the majority. In membranous nephropathy, renal amyloidosis, and endocapillary proliferative glomerulonephritis kappa light chain predominance was observed.

Serum creatinine was significantly higher in combined disease (NDRD+DN) in a study (1). Likewise, Mak* et al. *(5) and Lin* et al. *(8) did not find a difference in serum creatinine of isolated DN and NDRD. Despite finding a similar trend in our study, the difference was not statistically significant. Proteinuria was higher in patients with DN than those with NDRD. It indicates the progression of diabetes and predicts adverse renal outcomes (3,4,18).

Retinopathy was found in fewer members of group 1, while approximately half of them from the other two groups were affected. Wilfred* et al. *(4) ruled out the possibility of a selection bias affecting the result of finding a lower frequency of retinopathy in the NDRD group as all patients in their study underwent biopsy regardless of their fundus status and disease duration. Thus, the absence of retinopathy signals further investigations for non-diabetic glomerular diseases (24). Tone* et al. *(25) demonstrated 87% sensitivity and 93% specificity for the absence of retinopathy, whereas, according to Wong (26), the absence of retinopathy combined with hematuria and/or proteinuria ≥2 g/day was the most sensitive marker for NDRD, a strong indicator of biopsy. Even in our study, a significantly greater proportion of NDRD demonstrated hematuria. Sirisha* et al. *(3) presumed the active sediment in NDRD to be related to the occurrence of IgA nephropathy in NDRD.

The proportion of patients with low c3 and c4 levels and hypertension did not vary across the groups. Results from studies by Yaqub et al, (18) Soni* et al. *(7), and Matias* et al. *(27) were identical. However, few others (3,28) established systolic pressure as a predictor of DN. Liu* et al. *(29) detected fewer patients with uncontrolled HTN in NDRD; they linked this association to a higher dietary salt intake. Moreover, as nearly half of their patients were on RAAS blockade therapy during biopsy, they inferred that controlled BP could have prevented the progression of renal disease.

Therefore, considering the reversible nature of NDRD, early identification of certain clinical markers: hematuria, minimal retinopathy, and shorter duration of DM- as demonstrated in our study, help in achieving a better renal outcome because, being aware of the precise indications for renal biopsy in NDRD, permits an accurate diagnosis and prompt treatment. However, some limitations of our study include- studying a smaller sample from a single hospital- a bigger sample size and a multicenter study would better define the prevalence of NDRD. A further regression analysis would reveal the predictive value of each indicator highlighted in the study. Second, blinding was not followed, and third, electron microscopy was not done. Class I diabetic nephropathy is characterized only by diffuse glomerular basement membrane thickening and is diagnosed with the help of electron microscopy. As we intended to study NDRD in diabetic patients presenting with renal dysfunction, lack of EM leading to missing the changes of class I DN will not have an impact on the results of the study. However, the usage of electron microscopy will help correct the subtyping of non-diabetic renal disease and confirm the light microscopy and immunofluorescence findings. Hence, when considering renal biopsy in a diabetic patient, it would be wise to plan an electron microscopic examination as well. With the increasing ease of access to EM and EM becoming more affordable, it should be considered in all diabetic patients who are undergoing biopsy for suspicion of non-diabetic renal disease.

## Conclusion

In our study, significantly greater hematuria and lesser proteinuria were found in the NDRD group. In addition, a lesser duration of DM and negligible retinopathy should prompt a biopsy in diabetics for an early identification of NDRD. To conclude, an accurate and timely diagnosis would help clinicians offer appropriate therapy, thereby prolonging renal survival. 
